# On the performance analysis of a DCSK system under the pulse jamming environment

**DOI:** 10.1371/journal.pone.0201928

**Published:** 2018-08-14

**Authors:** Huu-Trien Khieu, Dang-Khanh Le, Binh Van Nguyen

**Affiliations:** 1 VMU College, Vietnam Maritime University, Hai Phong, Vietnam; 2 Institute of Research and Development, Duy Tan University, Da Nang 550000, Vietnam; 3 School of Electrical Engineering and Computer Science, Gwangju Institute of Science and Technology, Gwangju, Republic of Korea; Nanjing University of Information Science and Technology, CHINA

## Abstract

This paper considers the DCSK system under jamming environment. We consider both fast and slow switching pulse jamming (PJ). We derive approximations of the system bit error rates (BERs) in analytic expressions containing only well-known special functions, from which we show that for both jamming cases increasing the spreading factor can enhance the BER. In addition, we reveal that under the fast switching PJ, the BER does not depend on the jamming duty cycle *ρ* when *ρ* ≤ 0.5, however when *ρ* > 0.5, increasing its value degrades the BER. Moreover, we find out that under the slow switching PJ, increasing *ρ* may either enhance or degrade the BER.

## Introduction

Chaotic communications (CC) has recently been considered as a promising alternative to the conventional direct sequence spread-spectrum (SS) systems. The basic idea of CC is to replace pseudo-noise sequences by chaotic sequences, which can be directly generated by chaotic maps. Among various chaotic maps, logistic has been extensively exploited due to its simplicity and good performance [1]. Chaotic systems are generally categorized as coherent and non-coherent ones. In the former systems, chaotic synchronization plays a vital role in deciding the systems performance. However, designing an efficient chaotic synchronization scheme still remains as a challenging problem. As a result, research works on the coherent chaotic systems are very limited. On the other hand, in the latter systems, data recovery procedure is much simpler than that of the coherent counterparts since the chaotic synchronization is not required. Consequently, a lot of efforts have been devoted to design effective non-coherent modulation schemes and to find novel applications of non-chaotic coherent systems in reality [2].

The fundamental coherent and non-coherent modulation schemes are chaotic shift keying (CSK) and differential CSK (DCSK), respectively. The performances of chaotic systems with CSK and DCSK modulation schemes, referred as CSK and DCSK systems, over the additive white Gaussian noise (AWGN) channel are extensively investigated in [3]–[6]. It is shown that increasing the length of the spreading sequence improves the performance of the CSK system. On the other hand, enlarging the spreading factor beyond a certain point degrades the performance of the DCSK system. Recently, several advanced derivatives of the DCSK system have been proposed to improve the system performance, i.e. improved-DCSK (I-DCSK) [7], short reference-DCSK (SR-DCSK) [8], and noise reduction-DCKS (NR-DCSK) [9]. In addition, the emerging index modulation is also integrated into the DCSK system to improve the system’s data rate, energy efficiency, and spectral efficiency, i.e. permutation index-DCSK (PI-DCSK) [10] and commutation code index-DCSK (CCI-DCSK) [11]. Moreover, the DCSK system has also found applications in simultaneous wireless information and power transfer system [12].

Although the noise performances of coherent and non-coherent systems have been well studied [3]–[6], very little attention has been paid to tackle the effect of jamming on the performances of these systems. A summary of existing works on this subject is given in the Table 1. In [13], the performances of CSK and DCSK systems under the single-tone jamming (STJ) environment are analyzed. The authors show that the jamming frequency has little effect on the performance of a CSK system, however, the performance of a DCSK system is significantly degraded when the jamming frequency is an integer multiple of the bit frequency. In addition, the authors of [14] consider a CSK system under the pulse jamming (PJ) environment. It is pointed out that when the jamming is slow switching, increasing the jamming duty cycle may increase or decrease the system performance. However, when the jamming is fast switching the system performance is fully independent of the duty cycle. It is first noted that DCSK system is the fundamental and most studied non-coherent chaotic system. In addition, it is widely applied to other practical systems, i.e. multiple input—multiple output, cooperative, channel and network coding systems [15]–[17]. Secondly, the PJ is in fact a typical interference for wireless communication systems [18]. Consequently, the effects of the PJ on the performance of DCSK system is important and worth being investigated. Motivated by this fact, in this work, we will consider a DCSK system under the PJ environments. We shall derive approximations of the system bit-error-rates (BER) in analytic expressions which includes only well-known special functions, from which novel insights about the effect of the slow and fast switching PJ on the system performance will be carried out. In addition and importantly, from our results together with [13] and [14], comparisons of (i) the effects of the PJ on CSK and DCSK systems and (ii) the effects of the STJ and the PJ on a DCSK system will be discussed.

**Table 1:** A summary of existing works studying the anti-jamming performance of the CSK and the DCSK systems.

Jamming types	CSK modulation	DCSK modulation
**Single-tone jamming**	[13]	[13]
**Pulse jamming**	[14]	To be addressed in this paper

## 1 System and Jamming Models

### 1.1 System Model

The considered system consists of a pair of source-destination and a jammer. The DCSK scheme is employed for modulating and demodulating legitimate signals. In addition, the logistic map is exploited for generating chaotic sequences. A mathematical representation of the map is given by [1]

$$\begin{array}{c} x_{k+1}=1-2x_{k}^{2}, \end{array}$$ (1)

where *E* [*x*_*k*_] = 0, $E\;[x_{k}^{2}]=1/2$, and $\text{var}\;[x_{k}^{2}]=1/8$. For the modulation process, each bit duration is divided into two equal time slots. The first time slot is allocated for transmitting a reference chaotic sequence length *β*, called spreading factor. In the second time slot, either the reference sequence or its inverted version is transmitted depending upon the information bit. As a result the transmitted signal of the *l*^*th*^ bit, *b*_*l*_, can be expressed as

$$\begin{array}{c} s_{k}=\{\begin{array}{c} x_{k},\;\;\;\;\;\text{for}\;\;k=2\beta (l-1)+1,\cdots ,(2l-1)\beta \\ b_{l}x_{k-\beta },\;\;\text{for}\;\;k=(2l-1)\beta +1,\cdots ,2l\beta \end{array} \end{array}$$ (2)

While going through a channel to the destination, the transmitted signal *s*_*k*_ is affected by pulse jamming and additive white Gaussian noise (AWGN). The channel with jamming and AWGN is considered here so that we can solely focus on the effect of jamming on the system performance. This assumption has also been adopted in several related works, i.e. [13]–[14]. The joint effects of jamming and fading on the performance of the DCSK system are also interesting and will be considered in our future works. Finally, at the receiver side, the received signal is first correlated with its delayed version (delayed *β* samples), accumulated over a half bit duration, and passed through a threshold detector for deciding the transmitted bit.

### 1.2 Jamming Model

We consider the both fast switching PJ (FSPJ) and slow switching PJ (SSPJ). In addition, the jammer is assumed to turn on and off periodically. During the on time, the jamming signal can be considered as a zero-mean Gaussian variable with variance *P*_*j*_/*ρ* in the equivalent baseband discrete-time model, where *P*_*j*_ is the average power of the jammer [14].

The PJ is fast switching when its switching frequency is close to the bit frequency. Hence, it can be assumed that a fraction of *ρ* of every symbol is jammed. Let’s define λ = *ρ*2*β* as the period of time during which the jammer is on. In addition, for simplicity, we assume that λ is an integer and the jammer is turned on at the beginning of every symbol. Then, we can express the jamming signal during the *l*^*th*^ bit (symbol) period as follows

$$\begin{array}{c} j_{k}=\{\begin{array}{c} w_{k},\;\text{for}\;k=2(l-1)\beta +1,\cdots ,2(l-1)\beta +\lambda ,\\ 0,\;\;\;\;\text{for}\;k=2(l-1)\beta +\lambda +1,\cdots ,2l\beta , \end{array} \end{array}$$ (3)

where *w*_*k*_ is a zero-mean Gaussian variable with variance $\frac{P_{j}}{\rho }$.

On the other hand, the PJ is slow switching when its switching frequency is much lower than the bit frequency. In other words, the period during which the jammer is on or off is much longer than the bit duration. Consequently, the probability of a symbol being jammed is commonly approximated as *ρ* [14].

### 1.3 System Transmission

An equivalent baseband discrete-time model of the received signal at the receiver can be expressed as follows

$$\begin{array}{c} r_{k}=s_{k}+j_{k}+n_{k}, \end{array}$$ (4)

where *n*_*k*_ is the zero-mean AWGN with variance *N*_0_/2 and *j*_*k*_ denotes the jamming signal. Considering the *l*^*th*^ transmitted bit, the output of the correlator is given by

$$\begin{array}{cc} y_{l} & =\sum_{k=2(l-1)\beta +1}^{(2l-1)\beta }r_{k}r_{k+\beta }=\underset{\text{desired}\;\text{signal}}{\underset{︸}{A_{l}}}\\ & +\underset{\text{jamming}}{\underset{︸}{B_{1l}+B_{2l}+B_{3l}}}+\underset{\text{noise}}{\underset{︸}{C_{1l}+C_{2l}+C_{3l}+C_{4l}+C_{5l}}}, \end{array}$$ (5)

where

$$\begin{array}{cc} & A_{l}=\sum_{k=2(l-1)\beta +1}^{(2l-1)\beta }b_{l}x_{k}^{2}, \end{array}$$ (6)

$$\begin{array}{cc} & [\begin{array}{c} B_{1l}\\ B_{2l}\\ B_{3l} \end{array}]=\sum_{2(l-1)\beta +1}^{(2l-1)\beta }[\begin{array}{c} x_{k}j_{k+\beta }\\ b_{l}x_{k}j_{k}\\ j_{k}j_{k+\beta } \end{array}], \end{array}$$ (7)

$$\begin{array}{cc} & [\begin{array}{c} C_{1l}\\ C_{2l}\\ C_{3l}\\ C_{4l}\\ C_{5l} \end{array}]=\sum_{2(l-1)\beta +1}^{(2l-1)\beta }[\begin{array}{c} x_{k}n_{k+\beta }\\ b_{l}x_{k}n_{k}\\ j_{k}n_{k+\beta }\\ n_{k}j_{k+\beta }\\ n_{k}n_{k+\beta } \end{array}]. \end{array}$$ (8)

## 2 System Performance Analysis

Although the F-distribution based approach for deriving an exact BER of a DCSK system is presented in [6], its result is in a double-integral expression, from which it is challenging to reveal possible novel insights without doing numerical integration. On the other hand, the Gaussian approximation approach (GAA) can provide a simple closed-form approximation of the BER of a DCSK system. In addition, the approximation follows an exact one tightly with reasonable large values of the spreading factor. Hence, we will exploit the GAA to derive the BER of our considered system. According to the GAA, the output of the correlator (at the receiver) is assumed to follow the Gaussian distribution, and thus, the error probability of transmitting the *l*^*th*^ bit can be approximated as [13]

$$\begin{array}{cc} BER_{l} & =\mathrm{Pr}[b_{l}=1]\mathrm{Pr}[y_{l}\leq 0|b_{l}=1]+\mathrm{Pr}[b_{l}=-1]\mathrm{Pr}[y_{l}>0|b_{l}=-1]\\ & ≃\frac{1}{4}\text{erfc}(\frac{E[y_{l}|b_{l}=1]}{\sqrt{2\text{var}[y_{l}|b_{l}=1]}})+\frac{1}{4}\text{erfc}(\frac{-E[y_{l}|b_{l}=-1]}{\sqrt{2\text{var}[y_{l}|b_{l}=-1]}}), \end{array}$$ (9)

where Pr[⋅], *E*[⋅], and var[⋅] denote the probability, expectation, and the variance, respectively. Let’s first consider the FSPJ case, in which the mean and variance of the output of the correlator *y*_*l*_ can be derived by considering the following two cases: λ ≤ *β* and λ > *β*.

When λ ≤ *β* (and thus *ρ* ≤ 0.5), we have *B*_1*l*_ = *B*_3*l*_ = *C*_4*l*_ = 0. In addition, *B*_2*l*_ and *C*_3*l*_ can be rewritten as

$$\begin{array}{c} {}[\begin{array}{c} B_{2l}\\ C_{3l} \end{array}]=\sum_{2(l-1)\beta +1}^{2(l-1)\beta +\lambda }[\begin{array}{c} b_{l}x_{k}w_{k}\\ w_{k}n_{k+\beta } \end{array}]. \end{array}$$ (10)

On the other hand, when λ > *β* (and thus *ρ* > 0.5), *B*_1*l*_, *B*_2*l*_, *B*_3*l*_, *C*_3*l*_, and *C*_4*l*_ can be rewritten as

$$\begin{array}{c} B_{1l}=\sum_{2(l-1)\beta +1}^{(2l-3)\beta +\lambda }x_{k}w_{k+\beta }, \end{array}$$ (11)

$$\begin{array}{cc} & \left[\begin{array}{c} B_{2l}\\ C_{3l} \end{array}]=\sum_{2(l-1)\beta +1}^{(2l-1)\beta }[\begin{array}{c} b_{l}x_{k}w_{k}\\ w_{k}n_{k+\beta } \end{array}\right] \end{array}$$ (12)

$$\begin{array}{cc} & \left[\begin{array}{c} B_{3l}\\ C_{4l} \end{array}]=\sum_{2(l-1)\beta +1}^{(2l-3)\beta +\lambda }[\begin{array}{c} w_{k}w_{k+\beta }\\ n_{k}w_{k+\beta } \end{array}\right] \end{array}$$ (13)

From (6)–(8) and (10)–(13), we can show that for a general value of λ, $E\;[A_{l}]=b_{l}\beta E[x_{k}^{2}]$, *E* [*B*_*il*_] = 0, *E* [*C*_*jl*_] = 0, and

$$\begin{array}{cc} & \text{var}\;[A_{l}]=\beta \text{var}\;[x_{k}^{2}], \end{array}$$ (14)

$$\begin{array}{cc} & \text{var}\;[B_{1l}]=(\lambda -\beta )\frac{P_{j}}{\rho }\text{var}\;[x_{k}]u[\lambda -\beta ], \end{array}$$ (15)

$$\begin{array}{cc} & \text{var}\;[B_{2l}]=\min(\lambda ,\beta )\frac{P_{j}}{\rho }\text{var}\;[x_{k}], \end{array}$$ (16)

$$\begin{array}{cc} & \text{var}\;[B_{3l}]=(\lambda -\beta )\frac{P_{j}^{2}}{\rho^{2}}u\;[\lambda -\beta ], \end{array}$$ (17)

$$\begin{array}{cc} & \text{var}\;[C_{1l}]=\text{var}\;[C_{2l}]=\beta \frac{N_{0}}{2}\text{var}\;[x_{k}], \end{array}$$ (18)

$$\begin{array}{cc} & \text{var}\;[C_{3l}]=\min(\lambda ,\beta )\frac{P_{j}}{\rho }\frac{N_{0}}{2}, \end{array}$$ (19)

$$\begin{array}{cc} & \text{var}\;[C_{4l}]=(\lambda -\beta )\frac{P_{j}}{\rho }\frac{N_{0}}{2}u[\lambda -\beta ], \end{array}$$ (20)

$$\begin{array}{cc} & \text{var}\;[C_{5l}]=\beta \frac{N_{0}^{2}}{4}, \end{array}$$ (21)

where *i* = {1, 2, 3}, *j* = {1, ⋯, 5}, and

$$\begin{array}{c} u[\lambda -\beta ]=\{\begin{array}{c} 0,\;\;\text{for}\;\;\lambda \leq \beta ,\\ 1,\;\;\text{for}\;\;\lambda >\beta . \end{array} \end{array}$$ (22)

In addition, by exploiting the fact that the means of *x*_*k*_, *j*_*k*_, and the AWGN are all zero, we can readily prove that the covariance between any pairs of {*A*_*l*_, *B*_1*l*_, *B*_2*l*_, *B*_3*l*_, *C*_1*l*_, *C*_2*l*_, *C*_3*l*_, *C*_4*l*_, *C*_5*l*_} is zero. Consequently, we obtain

$$\begin{array}{cc} & E\;[y_{l}|b_{l}=1]=\beta E\;[x_{k}^{2}], \end{array}$$ (23)

$$\begin{array}{cc} & \text{var}\;[y_{l}|b_{l}=1]=\beta \text{var}\;[x_{k}^{2}]+\beta \text{var}\;[x_{k}](2P_{j}+N_{0})\\ & \;\;\;\;\;\;\;\;\;\;\;+\beta N_{0}(P_{j}+\frac{N_{0}}{4})+(\lambda -\beta )\frac{P_{j}^{2}}{\rho^{2}}u[\lambda -\beta ], \end{array}$$ (24)

$$\begin{array}{cc} & E[y_{l}|b_{l}=-1]=-E[y_{l}|b_{l}=1], \end{array}$$ (25)

$$\begin{array}{cc} & \text{var}\;[y_{l}|b_{l}=-1]=\text{var}\;[y_{l}|b_{l}=1]. \end{array}$$ (26)

Finally, replacing the results given in (23)–(26) into (9) gives the error probability of transmitting the *l*^*th*^ bit *BER*_*l*_. In addition, from the fact that *BER*_*l*_ does not depend on *l*, the whole system error probability *BER* will be the same as *BER*_*l*_ and is given as follows

$$\begin{array}{cc} BER≃ & \frac{1}{2}\text{erfc}\;[\frac{\sqrt{\beta }}{2}(\frac{1}{4}+2P_{j}+N_{0}+2N_{0}(P_{j}+\frac{N_{0}}{4})\\ & {+2P_{j}^{2}(\frac{2}{\rho }-\frac{1}{\rho^{2}})u[\lambda -\beta ])}^{-1/2}], \end{array}$$ (27)

from which we observe that

- When *ρ* ≤ 0.5, the system BER is independent of *ρ*.
- When *ρ* > 0.5, increasing its value degrades the system BER. The reason is that $\left(\frac{2}{\rho }-\frac{1}{\rho^{2}}\right)$ monotonically increases as *ρ* enlarges in (0.5, 1].
- Increasing the length of the chaotic sequence *β* can enhance the system BER.

From the above observation and [14], we see that the FSPJ has different effects on DCSK and CSK systems. Particularly, the BER of a CSK system is independent of the jamming duty cycle [14]. However, this only holds true for the DCSK system when the duty cycle is less than or equal to 0.5. On the other hand, when the duty cycle is larger than 0.5, increasing its value degrades the BER of the DCSK system.

By following the afore-presented procedure we can also derive the BER of the DCSK system under the SSPJ. After a similar manipulation like that for the case of FSPJ with GAA, one may obtain the system BER under the SSPJ as follows

$$\begin{array}{c} BER≃\frac{1-\rho }{2}\text{erfc}\;[\frac{\sqrt{\beta }/2}{\sqrt{\frac{1}{4}+N_{0}(1+\frac{N_{0}}{2})}}]\\ +\frac{\rho }{2}\text{erfc}\;[\frac{\sqrt{\beta }/2}{\sqrt{\frac{1}{4}+(\frac{2P_{j}}{\rho }+N_{0})+2(\frac{P_{j}^{2}}{\rho^{2}}+\frac{P_{j}}{\rho }N_{0}+\frac{N_{0}^{2}}{4})}}], \end{array}$$ (28)

which, together with [14], points out that the SSPJ has the same effects on DCSK and CSK systems. That is, increasing *β* can enhance the systems performance. In addition, increasing *ρ* may increase or decrease the systems performances. The reason is that although increasing *ρ* decreases the values of the *erfc*[⋅] functions, the factor *ρ*/2 being multiplied to the second *erfc*[⋅] function makes the BER’s trend uncertain.

## 3 Simulation Results

**Fig 1:**
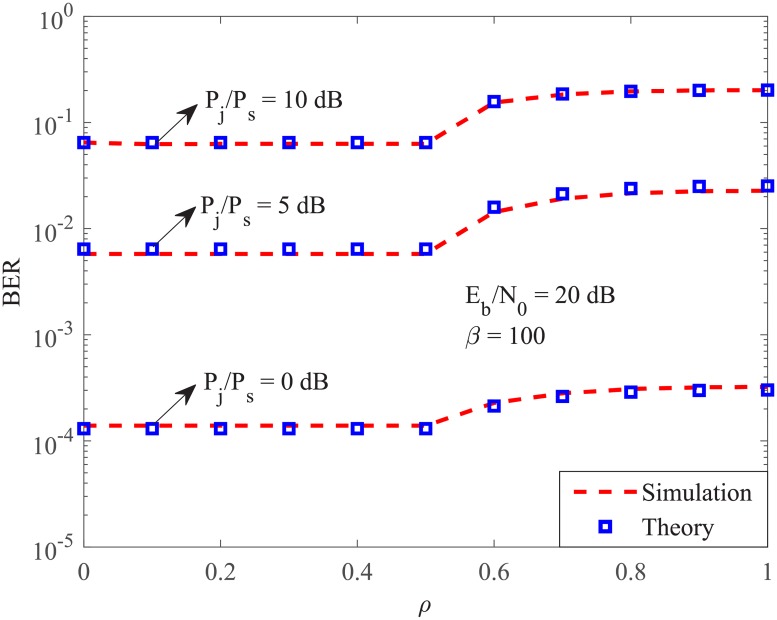
System BER versus *ρ* under the fast switching pulse jamming for several value of *P*_*j*_/*P*_*s*_.

In this section, simulated and analytic results are presented to verify our analyses. The BER versus the jamming duty cycle of the FSPJ is given in Fig 1. It is first shown that when *ρ* ≤ 0.5, the system BER is independent of *ρ*. In addition, when *ρ* > 5, increasing its value enlarges the BER. It is also noted that when *ρ* approaches 1, the increase of the BER is negligible. The reason is that the term $\frac{2}{\rho }-\frac{1}{\rho^{2}}$ given in (27) slowly converges to 1 as *ρ* tends to 1.

**Fig 2:**
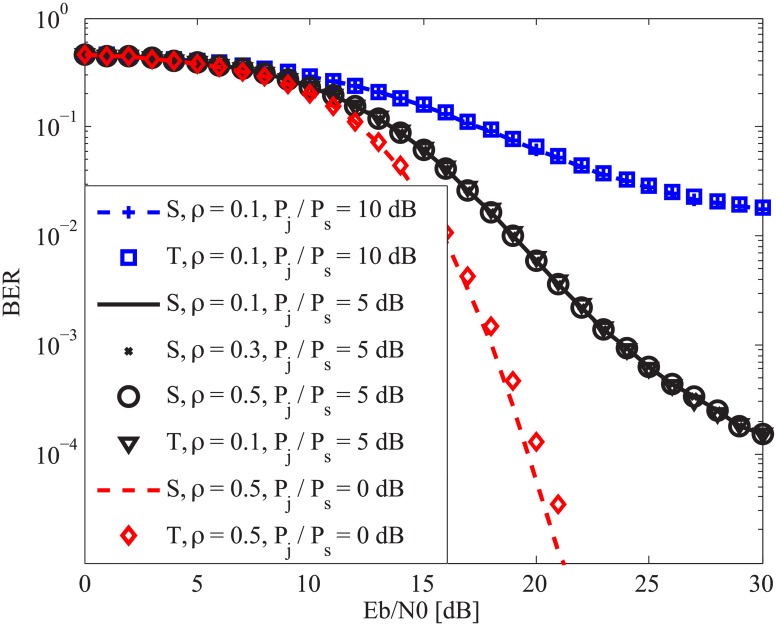
System BER versus *E*_*b*_/*N*_0_ for the case that the fast switching pulse jammer randomly turns on within a symbol duration with *β* = 100.

In Fig 2, we present the system BER versus *E*_*b*_/*N*_0_ for the case that the FSPJ randomly turns on within a symbol duration. Let *T*_*start*_ be the starting instant of the jammer which is a random integer between 2(*l* − 1)*β* and 2*lβ* − *χ*. *χ* is used here to make sure that every symbol is jammed. Depending upon the value of *T*_*start*_ we can have the following cases: only chaotic sequences are jammed, only information bearing sequences are jammed, two sequences of every symbol are jammed, and chaotic and information bearing sequences belonging to any two consecutive symbols are jammed. In the figure, S and T denote simulation and theory, respectively. It is shown that regardless of the value of *T*_*start*_, our findings about the effect of the FSPJ on the system performance still hold and our analytic results still follow the simulated ones tightly. In addition, we observe that increasing *P*_*j*_/*P*_*s*_ degrades the system performance, as expected.

**Fig 3:**
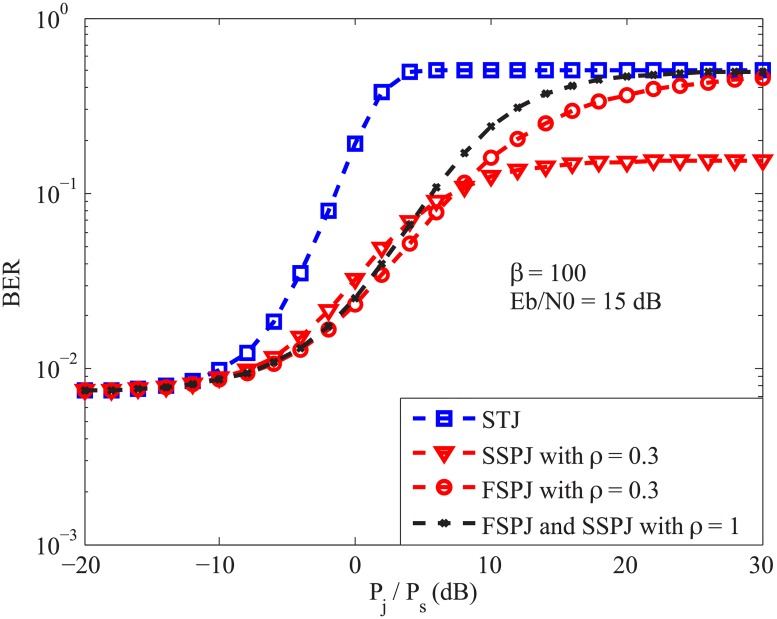
A comparison of system BERs under the fast switching pulse jamming, slow switching pulse jamming, and the single-tone jamming environments.

We illustrate the system BER versus *P*_*j*_/*P*_*s*_ under the STJ, FSPJ, and the SSPJ environments in Fig 3. The curve representing the system BER under the STJ is obtained by using the equation (73) given in [13]. The figure first shows that the STJ is the worst jamming type. Secondly, for the two PJs, the corresponding BERs stay more or less the same in the low region of *P*_*j*_/*P*_*s*_. However, when *P*_*j*_/*P*_*s*_ goes large, the FSPJ causes a much larger BER than that provided by the slow switching counterpart. The reason is that in the low region of *P*_*j*_/*P*_*s*_, the effect of jamming is dominated by that of the AWGN. On the contrary, in the high region of *P*_*j*_/*P*_*s*_, the effect of jamming becomes superior, and since under the FSPJ scenario it is most likely that every symbol is jammed, the corresponding error probability is larger than that caused by the SSPJ. Finally, the figure shows that as *ρ* tends to 1, the two BERs under the two PJs converge to each other and they approach the BER under the STJ in the high region of *P*_*j*_/*P*_*s*_.

**Fig 4:**
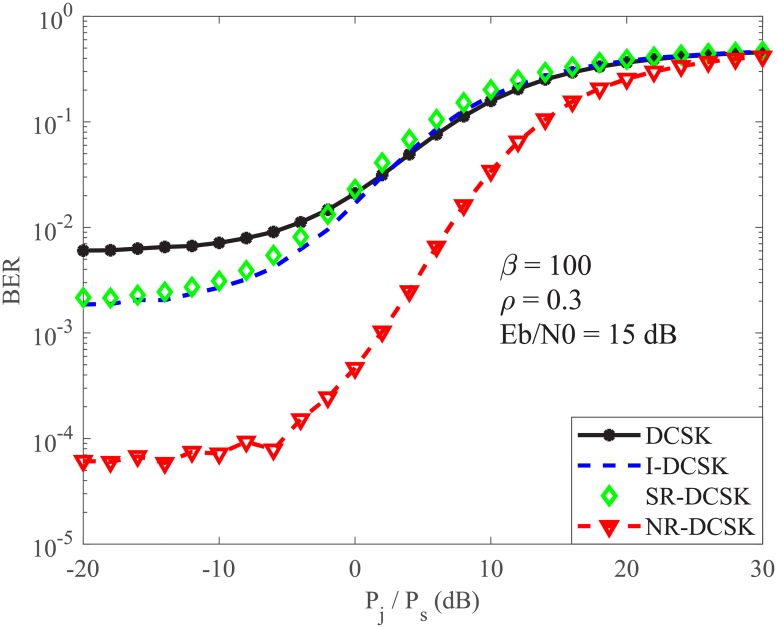
A comparison of the performance of the DCSK system and its derivatives under the fast switching pulse jamming environment.

Finally, we present a comparison of the performance of the DCSK system and its derivatives under the FSPJ environment in Fig 4. For the SR-DCSK scheme, for each bit duration, we use a reference sequence containing 50 chaotic samples. In addition, for the NR-DCSK scheme, for each bit duration, 5 chaotic samples are generated, and then, each chaotic sample is replicated 20 times to make a reference sequence of length *β* = 100. We observe that the DCSK scheme provides the worst performance, the NR-DCSK scheme provides the best performance, and the I-DCSK and the SR-DCSK give similar performance. In addition, it is noticed that the four scheme follow the same trend as the ratio *P*_*j*_/*P*_*s*_ increases.

## 4 Conclusion

In this work, we considered a non-coherent DCSK system under the slow and fast switching pulse jamming environments. We derived approximations of the system BERs in compact analytic expressions, from which we revealed that in general increasing the spreading factor can enhance the system anti-jamming performance. In addition, we showed that under the fast switching pulse jamming environment, the system BER is independent of the jamming duty cycle *ρ* when *ρ* ≤ 0.5, however when *ρ* > 0.5, the system BER is degraded when *ρ* increases. Moreover, we pointed out that under the slow switching pulse jamming scenario, increasing *ρ* may either improve or decrease the system BER. Furthermore, our simulation pointed out that the single-tone jamming is a more effective jamming type than the two pulse jamming counterparts. For our future works, we may consider the application of chaotic communications in physical layer security to enhance the secrecy performance of wireless communication systems [19]–[20].
